# Photometric Evaluation of Long-term Changes in Breast Shape after Breast Augmentation and Vertical Mammaplasty

**DOI:** 10.1097/GOX.0000000000001844

**Published:** 2018-06-18

**Authors:** Eric Swanson

**Affiliations:** Private practice, Leawood, Kansas.

## Abstract

**Background:**

Little information is presently available regarding the long-term effects of breast augmentation and mammaplasties on breast shape. Existing studies typically use 1-dimensional measurements and mean follow-up times seldom exceed 1 year.

**Methods:**

Twenty women were studied: breast augmentation (n = 5), mastopexy (n = 5), augmentation/mastopexy (n = 5), and breast reduction (n = 5). For mammaplasties, a vertical method using a medial pedicle and intraoperative nipple siting was used in all cases. A 2-dimensional measurement system was used, with computer-assisted matching of lateral photographs of the right and left breasts. Measurements were evaluated at 3 times: before surgery, 1 year after surgery, and 10 years after surgery.

**Results:**

Breast implants significantly increased breast projection and upper pole projection. The nipple level was unchanged. The lower pole level dropped. These changes were preserved at 10 years. Vertical mastopexy provided a modest increment in breast projection and upper pole projection. The nipple level and lower pole level were raised significantly. Augmentation/mastopexy boosted breast projection and upper pole projection, and also elevated the nipple and lower pole level. Breast reduction changes were similar to mastopexy, but with greater elevation of nipple level and lower pole level, which were usually lower to start with. Implants increased upper pole convexity. Nipple overelevation was avoided by intraoperative nipple siting just below the breast apex.

**Conclusions:**

These measurements provide new information regarding the long-term effects of breast augmentation and vertical mammaplasties. This information may be used by plastic surgeons in procedure selection and patient counseling.

## INTRODUCTION

Evaluation of long-term changes in breast morphology has been limited by a lack of objective measurements. Existing studies typically use 1-dimensional measurements made with a tape measure or ruler.^1–12^ Three-dimensional systems and magnetic resonance imaging have also been used.^13–15^ However, these studies have not evaluated long-term results. Most publications include patients with a mean follow-up time of approximately 1 year or less.^3,4,6,8,10–15^ Reus and Mathes^1^ measured surface distances an average of 4.7 years (range, 2.3–7.5 years) after reduction mammaplasties. Bouwer et al.^16^ studied patients 10 years after breast reductions but used a subjective scoring method; there were no measurements. The literature does not include 10-year follow-up using an objective and reliable measurement device to evaluate and compare cosmetic breast procedures, including breast reduction. This study was undertaken to remedy this deficiency in our knowledge base.

A 2-dimensional measurement system may be used to evaluate breast shape changes after surgery and is reported separately.^17^ This method has been used to evaluate and compare short-term changes (mean follow-up, 10 months) in breast morphology after breast augmentation, mastopexy, augmentation/mastopexy, and breast reduction using the vertical technique.^18^ This measurement system has also been used to assess published mammaplasty methods,^19^ and to compare vertical and inverted-T breast reductions.^20^

## PATIENTS AND METHODS

### Patients

This retrospective study evaluated standardized photographs of women undergoing breast augmentation, mastopexy, augmentation/mastopexy, and breast reduction. All procedures were performed by the author in the same state-licensed ambulatory surgery center. The study was determined to be exempt by Chesapeake Institutional Review Board, accredited by the Association for the Accreditation of Human Research Protection Programs, Inc.

Because the same vertical dissection and medial pedicle was used for all mammaplasties, any distinction between mastopexy and reduction is arbitrary. If the resection weight was ≥ 300 g for at least 1 breast, the operation was labeled a breast reduction.^15^

Eligibility criteria included (1) bilateral surgery; (2) long-term follow-up (mean, 10 years; range, 8–14 years); (3) photographs available; (4) no additional breast surgery; and (5) weight stability (ie, no weight change > 20 lbs.). Breast reconstructions were excluded.

### Surgery

Breast augmentation was performed using a supra-inframammary approach (incision 0.5–1.0 cm superior to the existing inframammary crease)^21^ and subpectoral implant placement in all patients. Nine women received saline implants: 1 patient was treated with silicone gel implants.

A vertical mammaplasty with intraoperative nipple siting and a medially based pedicle were used exclusively. A single-stage “all seasons” augmentation/mastopexy was performed^22^; no patients were staged for surgery.

### Photographs

All patients were photographed using a Nikon (Nikon Corp., Tokyo, Japan) digital single lens reflex camera and the same 60 mm lens. The same blue background, focal distance, and lighting were used for all photographs, which were taken in the same examining room. Patients were positioned with their arms to their sides. A ruler was included in 1 of the photographs to allow calibration.

### Measurements

**Fig. 1.**
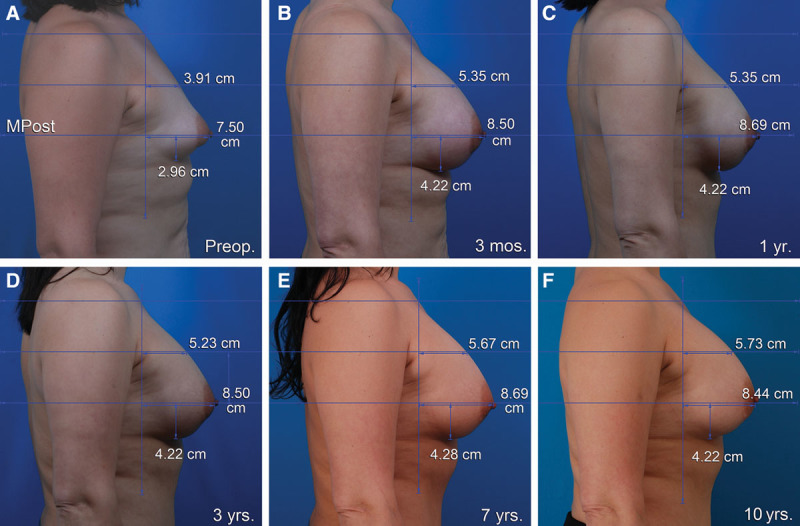
This 28-year-old woman underwent a breast augmentation and liposuction of the abdomen, flanks, and inner thighs. A 450 cc implant was used for the right breast (Mentor Moderate Plus Profile smooth, round silicone gel implants, Santa Barbara, Calif.). One year later, she had an abdominoplasty. She is seen before (A), 3 months after (B), 1 year after (C), 3 years after (D), 7 years after (E), and 10 years after her breast augmentation (F). The nipple level is unchanged. The lower pole level dropped 1.3 cm 3 months after surgery. Its position remains unchanged 10 years later. Breast projection and upper pole projection are preserved. The upper pole contour is convex. MPost, plane of maximum postoperative breast projection.

**Fig. 2.**
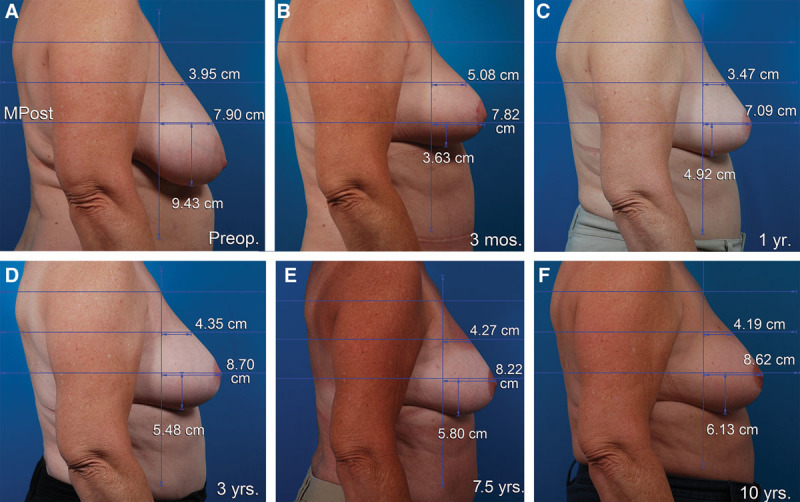
This 55-year-old woman underwent a vertical breast reduction using a medial pedicle. The right resection weight was 360 g. She had a simultaneous abdominoplasty and liposuction of the abdomen and flanks. She is seen before (A), 3 months after (B), 1 year after (C), 3 years after (D), 7.5 years after (E), and 10 years after surgery (F). The nipple position remains at the apex 10 years after surgery. The lower pole level is elevated 4.5 cm 1 year after surgery. Breast projection and upper pole projection are maintained 10 years after surgery. The breast upper pole contour is linear at 1 year and 10 years, with a convexity ratio of 0.49.

**Fig. 3.**
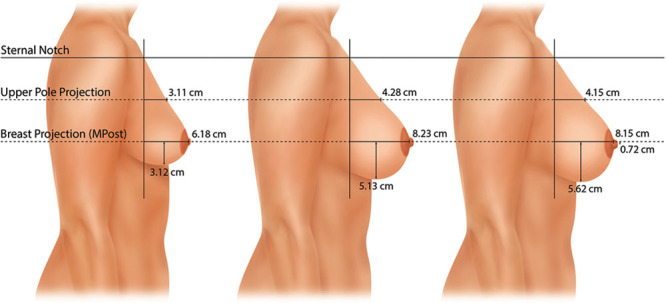
This mammograph represents the mean measurements for the patients undergoing breast augmentation. The right breast is depicted before surgery, 1 year after surgery, and 10 years after surgery. The lower pole drops 2 cm 1 year after surgery and another 0.5 cm at 10 years. The nipple position remains unchanged. Both breast projection and upper pole projection are significantly increased (2 cm and 1 cm, respectively).

**Fig. 4.**
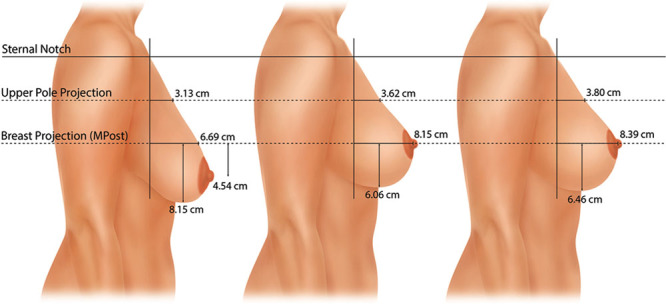
This mammograph represents the mean measurements for the patients undergoing vertical mastopexy. The right breast is depicted before surgery, 1 year after surgery, and 10 years after surgery. The lower pole is elevated 2.1 cm at 1 year but drops 0.4 cm between 1 and 10 years. The nipple position is corrected, with no sign of overelevation. Both breast projection and upper pole projection are increased. Breast convexity (upper pole projection/breast projection) measured 0.44 1 year after surgery and 0.45 10 years after surgery, reflecting a slightly concave profile.

**Fig. 5.**
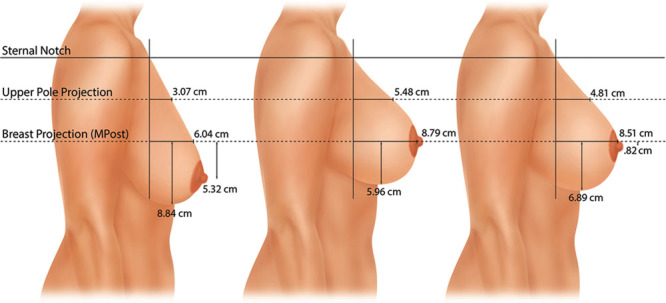
This mammograph represents the mean measurements for the patients undergoing vertical augmentation/mastopexy. The right breast is depicted before surgery, 1 year after surgery, and 10 years after surgery. The lower pole is elevated 3 cm but drops about 1 cm in long-term follow-up. At 10 years, the lower pole level remains 2 cm higher than its preoperative level. Both breast projection and upper pole projection are substantially increased. Breast convexity measured 0.62 at 1 year and 0.57 at 10 years.

**Fig. 6.**
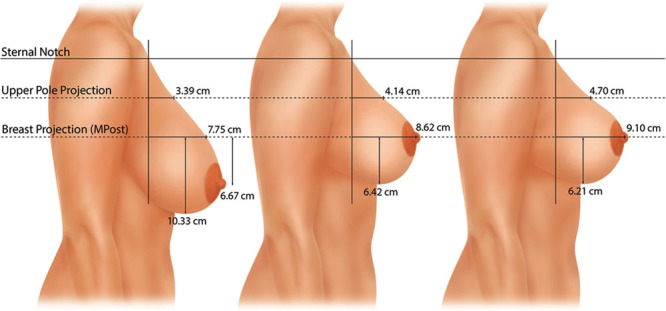
This mammograph represents the mean measurements for the patients undergoing vertical breast reduction. The right breast is depicted before surgery, 1 year after surgery, and 10 years after surgery. The nipple position is corrected, remaining at the level of the breast apex with no sign of overelevation. The lower pole is elevated 3.9 cm, with little change in the lower pole level between 1 year and 10 years. Breast projection and upper pole projection are slightly increased compared with preoperative values and this difference is maintained at 10 years. The upper pole contour is almost linear with a convexity ratio of 0.52.

Measurements were made on the lateral photographs. These dimensions included: (1) upper breast projection; (2) breast projection; (3) lower pole level; and (4) nipple level.^17^ The Canfield 7.4.1 Mirror Imaging software (Canfield Scientific, Fairfield, N.J.) was used to exactly match the orientation and size of the before-and-after photographs (Figs. 1, 2). The mean measurements for each group before surgery, 1 year after surgery, and 10 years after surgery are presented as mammographs in Figures 3–6. To conserve Journal space, only the right breast measurements are shown, and only 3 time points are illustrated.

### Statistical Analysis

Statistical analyses were performed using SPSS for Windows version 25.0 (SPSS, Inc., Chicago, Ill.). Independent *t* tests were used to compare means between 2 groups, and 1-way analyses of variance were used to compare means across more than 2 groups. A sample size calculation indicated that 20 patients and 4 groups would provide 80% power to detect a large treatment difference (*f* = 0.90). Paired *t* tests were used to compare preoperative measurements with 1-year and 10-year measurements. Tukey HSD post hoc comparisons were used to compare the group means when the analysis of variance was significant. A value of *P* < 0.05 was considered significant.

## RESULTS

Each patient group consisted of 5 women, for a total of 20 patients (Table 1). The mean patient age was 41 years (range, 24–57 years). Patients undergoing mastopexy were significantly older, on average, than the other patients (*P* = 0.001). The mean follow-up time for all patients was 10.8 years (range, 8–14 years). The mean breast implant volume for breast augmentation was 420 cc for the right breast and 414 cc for the left breast. The mean implant volume for augmentation/mastopexy was 361 cc on both sides. The mean resection weights for women undergoing augmentation/mastopexy (right, 85 g; left, 60 g) were lower (although not significantly) than for mastopexy patients (right, 169 g; left, 139 g).

**Table 1.**
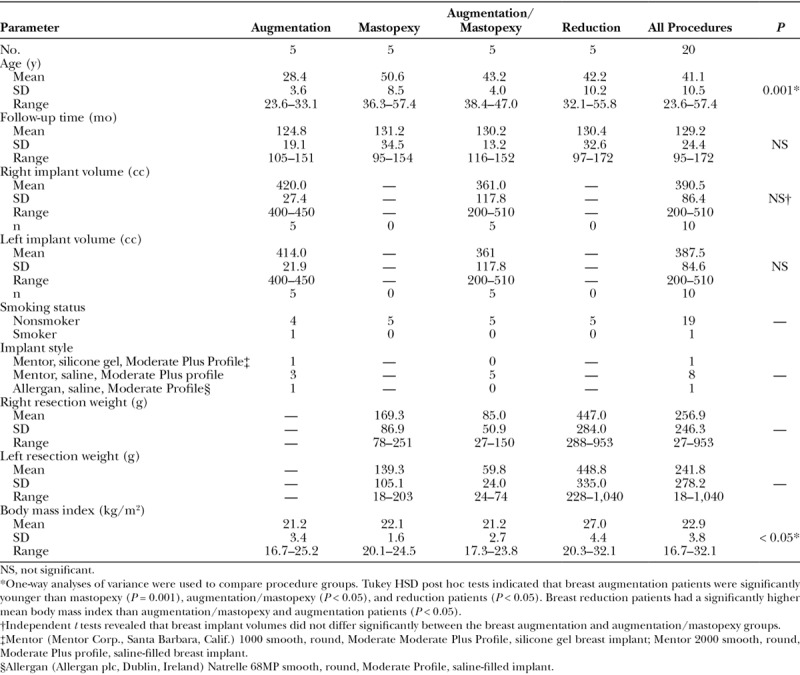
Data for 20 Patients Undergoing Breast Augmentation and Vertical Mammaplasties

### Breast Augmentation

Figure 1 depicts a breast augmentation patient at various times after surgery up to 10 years. Figure 3 is a mammograph representing mean measurements before surgery, 1 year after surgery, and 10 years after breast augmentation. The nipple level did not change significantly after breast augmentation. The lower pole level dropped significantly after surgery on both sides (*P* < 0.05). One year after surgery, the lower pole level was 2.0 cm lower for the right breast. Ten years after surgery, it dropped another 0.5 cm, on average (Fig. 3). Breast projection was increased at 1 year (right, 2.1 cm; left, 1.8 cm). This increase was maintained 10 years after surgery (right, 2.0 cm; left, 1.8 cm). Similarly, upper pole projection was increased at 1 year (right, 1,2 cm; left, 0.9 cm). Upper pole projection decreased slightly (but not significantly) at 10 years (Table 2).

**Table 2.**
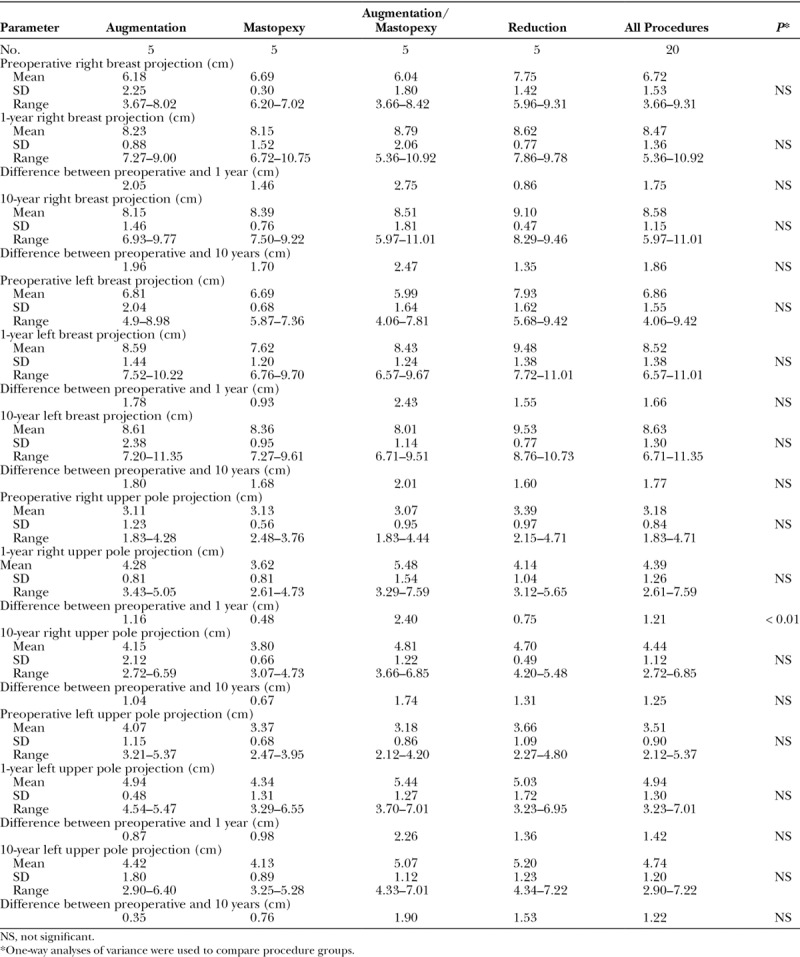
Breast Projection and Upper Pole Projection Before and After Breast Procedures

The right breast convexity (upper pole projection/breast projection) measured 0.53 on the right and 0.58 on the left 1 year after surgery. At 10 years, these ratios were 0.51 on both sides, reflecting a slightly convex profile.

### Vertical Mastopexy

Figure 4 is a mammograph that shows mean values for the right breast before, 1 year, and 10 years after vertical mastopexy. On average, the preoperative right nipple level was 4.5 cm below the level of maximum postoperative breast projection (MPost) and the left nipple was located 3.4 cm below this plane. After vertical mastopexy, the right nipple was elevated to the breast apex without overelevation (Fig. 4). The difference in nipple level was highly significant (*P* < 0.001) at 1 and 10 years, with no significant change in nipple level between 1 and 10 years. The lower pole level was also significantly elevated at 1 year (right, 2.1 cm; left, 2.2 cm). Between 1 and 10 years, the lower pole level settled 0.4 cm on the right and 0.9 cm on the left. At 10 years, the nipples remained significantly above their preoperative level (*P* < 0.05).

At 1 year, breast projection was increased 1.5 cm on the right and 0.9 cm on the left. At 10 years, breast projection remained 1.7 cm greater on both sides, significantly greater than before surgery (*P* < 0.01). Upper pole projection increased 0.5 cm on the right and 1.0 cm on the left at 1 year. At 10 years, the mean increments were 0.7 and 0.8 cm.

The right breast convexity (upper pole projection/breast projection) measured 0.44 1 year after surgery on the right and 0.57 on the left. These measurements were 0.45 and 0.49, respectively, at 10 years, reflecting a slightly concave profile (Fig. 4).

### Vertical Augmentation/Mastopexy

The mean right nipple level was 5.3 cm below the MPost plane before augmentation/mastopexy (Fig. 5), and the mean left nipple position was 4.8 cm below this plane. One year after surgery, the nipple level was at the level of the breast apex without overelevation on both sides. Ten years after surgery, the nipple was located 0.8 cm below the MPost plane on the right and 0.9 cm below this plane on the left. Nipple elevation was highly significant (*P* < 0.001) at both follow-up times. At 1 year, the right lower pole level was lifted 2.9 cm on the right and 2.5 cm on the left. At 10 years, these changes were 2.0 cm on the right and 1.5 cm on the left, indicating about 1 cm of settling on each side, although the lower pole levels remained significantly higher (*P* < 0.01) than the preoperative levels.

Breast projection increased 2.8 cm on the right and 2.4 cm on the left at 1 year. At 10 years, these increases were 2.5 cm and 2.0 cm. Upper pole projection increased 2.4 cm at 1 year on the right and 2.3 cm on the left. At 10 years, these increments had decreased to 1.7 cm and 1.9 cm, respectively.

Breast convexity measured 0.62 and 0.64 1 year after surgery versus 0.57 and 0.63 10 years after surgery, indicating that upper-pole convexity persisted long-term.

### Breast Reduction

The mean preoperative nipple levels were 6.7 cm below the MPost plane on the right side and 6.3 cm below this plane on the left side (Fig. 6). One year after surgery, the right nipple was located at the breast apex; the left nipple level was 0.5 cm overelevated, on average. At 10 years, the right nipple position was unchanged; the left nipple was 0.7 cm overelevated. These changes were significant at both time points on both sides (*P* < 0.05). The right lower pole was elevated 3.9 cm at 1 year on the right and 4.3 cm on the left. There was no significant settling of the lower poles between 1 and 10 years.

At 1 year, the right breast projection increased 0.9 cm and the left breast projection increased 1.6 cm. At 10 years, these increases measured 1.4 cm and 1.6 cm. These changes were not significant at either time point. Upper pole projection increased 0.8 cm on the right and 1.4 cm on the left at 1 year. At 10 years, these increases were 1.3 and 1.5 cm, respectively. This change was significant at 10 years (*P* < 0.05) but not at 1 year.

Breast convexity at 1 year was 0.48 on the right and 0.53 on the left. These ratios measured 0.52 and 0.55 at 10 years.

### Complications

None of the patients experienced systemic complications. No patient required a return to the operating room. One breast reduction patient had a small hematoma that was aspirated once in the office, and another reduction patient underwent a revision of dog ears in the office under local anesthesia. There were no implant-related complications.

## DISCUSSION

### Measuring Device

Tape measurements may be affected by interoperator error.^23^ When measuring the distance from the nipple to the IMF, the skin is subjected to a variable degree of stretch, increasing the likelihood of intrarater and interrater variability.^23^ Moreover, when a vertical method is used, the inframammary fold (IMF) level is elevated after surgery.^24^ Unlike the sternal notch, the IMF is not a constant landmark. Patients do not inquire about the nipple-IMF distance, which is ideally longer than the 5-cm vertical limb length used for Wise pattern mammaplasties^6,18^ in an (often unsuccessful^19^) effort to avoid nipple overelevation. Patients are more interested in whether the breast will sag again with time.

Three-dimensional systems have their own limitations.^17,25,26^ There is considerable subjectivity in identifying breast borders and matching subsequent images.^17^ Imaging the lower pole of the breast is a challenge, especially in large and ptotic breasts.^27–30^ Steen et al.^25^ found that 3-D measurements of base width and nipple-to-IMF distance did not correlate well with direct measurements because the landmarks are obscured.

Volume measurements are made difficult by the lack of a known boundary between the breast and chest wall.^17,26^ Computed topography or magnetic resonance imaging can be used to visualize this border,^17,26^ but these are expensive and inconvenient tests, and the patient is usually positioned supine or prone,^31,32^ defeating the purpose of shape analysis.^17^ The IMF is difficult to visualize, hidden on the frontal view, and overlapped by a ptotic breast.^17^ Many operators who have a 3-D system still rely on 1-dimensional surface measurements.^10,11,14^

The nipple is traditionally used as a landmark, but its position does not provide information regarding the shape and level of the breast mound.^33^ Breast shape and nipple position are 2 different parameters and are best considered separately.^33^

To accurately and objectively study morphologic changes, a 2-dimensional system is needed. Such a system is not too simple (ie, 1-dimensional measurements), yet not too complicated (3-D systems). It may be used to study existing lateral photographs, making retrospective studies possible, even on published photographs.^19^ There is no need for an expensive photographic setup. The reference plane is the level of maximum postoperative breast projection.^17^ The lower pole level, which is not hidden and easy to measure, replaces the IMF as a landmark. Breast area is a surrogate for volume. The chest wall component is the same when comparing postoperative and preoperative lateral images; there is no need to identify the breast/chest wall boundary. When preoperative area measurements are subtracted from postoperative area measurements, this contribution cancels out.^17^ There is no need for a virtual chest wall template.^17^

### Existing Studies

Hall-Findlay^5^ reported that the distance from the sternal notch to the nipple increased 2 cm after augmentation and augmentation/mastopexy. There was minimal elongation after mastopexy (0.5 cm) and reduction (0.3 cm). She also found that breast implants lower the lower pole and IMF, regardless of the surgeon’s efforts to preserve the fold and its ligamentous attachments. These findings were confirmed by the author’s previously published measurement studies.^18,22,24^ It is not clear that efforts to fix the IMF level are effective, especially in the long term.^34^

Several studies have evaluated short-term changes in breast dimensions after breast reduction. Reus and Mathes^1^ reported an elongation of the lower pole, measured from the inferior margin of the areola to the IMF, after a Wise pattern inferior pedicle breast reduction despite limiting the vertical limb to 5 cm. The distance from the midclavicle to the nipple did not change. Consequently, the nipple/areola was displaced superiorly on the breast mount after surgery. Similarly, Ahmad and Lista^5^ found that the nipple moved up on the breast mound after a vertical breast reduction, stimulating these surgeons to situate the nipple a little lower, with the superior areolar margin at the level of the IMF rather than the nipple itself. These authors found that the distance from the areola to the IMF shortens between 5 days after surgery and 4 years after surgery.^5^ Other investigators have found that that length of the vertical limb either stayed the same or increased after a breast reduction.^2,4^ This variability underscores the limitations of this surface measurement in assessing surgical results.

Changes after a vertical mastopexy are known to include a modest increase in upper pole projection and breast projection (0.5 cm and 1.2 cm, respectively), and elevation of the lower pole and nipple level.^18^ Changes after a breast reduction are similar. Indeed, if a vertical technique is used, the 2 operations are identical, differentiated only by the resection weights.

Patient surveys reveal that women prefer upper pole convexity.^35^ This appearance is most effectively produced by breast implants.^18^ Measurements reveal that breast autoaugmentation is ineffective.^19^ Breast implants are less prone to deformation^36^ and hold their shape more reliably than breast tissue, aided by capsular contraction.^18^

### Clinical Relevance

There are practical benefits to having long-term data. Patients should be informed that a breast augmentation does not “take up the slack.”^18^ In fact, the lower pole level is lower after the operation and gradually descends over time, although the nipple level tends to be static.

This study confirms earlier findings demonstrating the effectiveness of a vertical mastopexy and reduction in lifting the nipple and breast mound.^18^ The author determines the nipple level in surgery after creation of the breast mound.^18^ The nipple is sited slightly below the breast apex to avoid nipple overelevation, which is a common problem associated with the Wise pattern and inferior pedicle.^19^ Parenchymal resection (not just skin) avoids a persistent lower pole bulge that may require secondary correction.^37^

Augmentation/mastopexy combines the attributes of breast augmentation and mastopexy.^22,37^ The elevating effect of the mastopexy tends to overcome the lower-pole-lowering effect of an implant. The findings are summarized below:

### Breast Augmentation

1. The nipple position is not changed after breast augmentation.
2. Breast projection and upper pole projection are increased, and these increments remain stable over time.
3. The lower pole level drops after surgery and continues to settle gradually.

### Mastopexy

1. A vertical mastopexy produces a modest increase in breast projection and upper pole projection.
2. The nipple and lower pole level are elevated.
3. The lower pole settles gradually.
4. The upper pole contour is slightly concave.

### Augmentation/Mastopexy

1. More breast projection and upper pole projection are possible compared with a mastopexy without implants.
2. The nipple and lower pole level are elevated.
3. The lower pole settles over time.
4. Convexity of the upper pole is maintained.

### Reduction

1. Changes are similar to a mastopexy but with greater lift of the nipple and lower pole level. These changes persist long-term.

### Limitations of the Study

This retrospective study is limited by a small number (n = 20) of nonconsecutive patients. Cosmetic breast patients are notorious for not keeping long-term follow-up appointments, especially for research purposes.^38^ The findings are applicable only to vertical mammaplasties. The comparisons do not take into account changes in the breast from aging. There are no untreated controls for comparison.

### Strengths of the Study

Because the procedure is bilateral, 20 women provided 40 breasts for measurements at multiple time points. Weight-stable patients were treated by 1 surgeon using the same method, avoiding the influence of confounding variables.

## CONCLUSIONS

A 2-dimensional measurement system allows quantitative evaluation of breast shape changes over time, providing long-term data on which to base clinical decisions and inform patients regarding the longevity of the results.

## ACKNOWLEDGMENTS

The author thanks Jane Zagorski, PhD, for statistical analyses; Christina Engel, RT, for data collection; and Gwendolyn Godfrey for illustrations.
